# Adrenalectomy Reduces the Risk of Vertebral Fractures in Patients With Mild Autonomous Cortisol Secretion

**DOI:** 10.1210/clinem/dgaf227

**Published:** 2025-04-11

**Authors:** Valentina Morelli, Vittoria Favero, Sofia Frigerio, Carmen Aresta, Flavia Pugliese, Antonio Stefano Salcuni, Alessandro Risio, Cristina Eller-Vainicher, Serena Palmieri, Elisa Cairoli, Sabrina Corbetta, Giovanna Mantovani, Alfredo Scillitani, Iacopo Chiodini

**Affiliations:** Endocrinology Department of Endocrine and Metabolic Diseases, IRCCS Istituto Auxologico Italiano, 20149 Milan, Italy; Department of Medical Biotechnology and Translational Medicine, University of Milan, 20122 Milan, Italy; Unit of Endocrinology, ASST Grande Ospedale Metropolitano Niguarda, 20163 Milan, Italy; Unit of Endocrinology, Fondazione IRCCS Cà Granda-Ospedale Maggiore Policlinico, 20122 Milan, Italy; Unit of Endocrinology, ASST Grande Ospedale Metropolitano Niguarda, 20163 Milan, Italy; Unit of Endocrinology, “Casa Sollievo della Sofferenza Hospital” IRCCS, 71013 San Giovanni Rotondo, Foggia, Italy; Unit of Endocrinology and Metabolism, University-Hospital S. Maria Della Misericordia, 33100 Udine, Italy; Department of Medical Biotechnology and Translational Medicine, University of Milan, 20122 Milan, Italy; Unit of Endocrinology, Fondazione IRCCS Cà Granda-Ospedale Maggiore Policlinico, 20122 Milan, Italy; Unit of Endocrinology, Fondazione IRCCS Cà Granda-Ospedale Maggiore Policlinico, 20122 Milan, Italy; Endocrinology Department of Endocrine and Metabolic Diseases, IRCCS Istituto Auxologico Italiano, 20149 Milan, Italy; Endocrinology Department of Endocrine and Metabolic Diseases, IRCCS Istituto Auxologico Italiano, 20149 Milan, Italy; Department of Biomedical, Surgical and Dental Sciences, University of Milan, 20122 Milan, Italy; Unit of Endocrinology, Fondazione IRCCS Cà Granda-Ospedale Maggiore Policlinico, 20122 Milan, Italy; Department of Clinical Sciences and Community Health, University of Milan, 20122 Milan, Italy; Unit of Endocrinology, “Casa Sollievo della Sofferenza Hospital” IRCCS, 71013 San Giovanni Rotondo, Foggia, Italy; Endocrinology Department of Endocrine and Metabolic Diseases, IRCCS Istituto Auxologico Italiano, 20149 Milan, Italy; Department of Medical Biotechnology and Translational Medicine, University of Milan, 20122 Milan, Italy

**Keywords:** mild autonomous cortisol secretion, bone mineral density, vertebral fracture

## Abstract

**Context:**

Mild autonomous cortisol secretion (MACS) is associated with increased risk of vertebral fractures (VFx).

**Objective:**

The aim was to investigate impact of recovery from MACS on bone health remains unclear.

**Methods:**

Retrospective intervention study (Study 1): 53 patients with MACS were followed for 35.2 ± 18.6 months; 31 patients underwent surgery (Study 1-Group A, 74.2% women, age 63 years [57-67]), while 22 patients received conservative treatment (Study 1-Group B, 45.5% women, age 64 years [61-72]). Prospective randomized study (Study 2): Fifty-one outpatients with MACS were randomly assigned to either adrenalectomy (Study 2-Group A, 21 patients, 67% women, age 63 [56.5-72.5]) or conservative approach (Study 2-Group B, 28 patients, 78% women, age 69 [61-73]) and were followed for 24 months.

**Methods:**

MACS was diagnosed in patients with adrenal incidentalomas (AIs) >1 cm and cortisol after the 1-mg dexamethasone suppression test ≥1.8 µg/dL (50 nmol/L). At baseline and at the end of follow-up we assessed calcium–phosphorus metabolism, bone mineral density (BMD) at the lumbar spine (LS), total hip (TH), and femoral neck (FN) using dual-energy X-ray absorptiometry, and the presence of VFx.

**Results:**

Study 1: At the end of the follow-up, Study 1-Group B showed an increased incidence of VFx (n = 11, 50%) than Study 1-Group A (n = 3, 9.7%, *P* < .005). In both groups, BMD at LS, FN, and TH was comparable between baseline and the end of follow-up. Study 2: After 24 months in Study 2-Group A, but not in Study 2-Group B, calcium and phosphorus levels increased compared with baseline (*P* = .03 and *P* = .04, respectively). At the end of follow-up, BMD remained stable across both groups, but Study 2-Group B showed a significantly higher incidence of VFx (n = 7, 25%) than Study 2-Group A (n = 1, 4.8%, *P* = .04).

**Conclusion:**

In patients with AI and MACS, adrenalectomy significantly reduces the risk of VFx.

Nowadays, “mild autonomous cortisol secretion (MACS)” is the term proposed by the European Society of Endocrinology (ESE) in collaboration with the European Network for the Study of Adrenal Tumors (ENSAT) to define the condition of biochemically evident cortisol excess in patients without the typical clinical features of cortisol excess (1). In the recent years, MACS has been receiving more and more attention since it has a non-negligible prevalence (ie, up to 2% in adults), being present in up to 50% of patients with adrenal incidentalomas (AIs), which, in turn, are diagnosed in up to 7% of subjects above 60 years of age (2). Moreover, MACS is considered an important condition since it may lead to cardiometabolic consequences (3). Importantly, data from intervention studies have suggested that the recovery from MACS ameliorates glucometabolic control and blood pressure in patients with AI (4, 5).

Beside the cardiometabolic consequences, in the past, several studies showed that even the less severe form of hypercortisolism was associated with bone fragility (6) and that surgery was beneficial in these patients (7). However, data on bone involvement in patients with MACS, as defined on the basis of the ESE-ENSAT guidelines, are scarce. Indeed, the new criteria suggest that, in patients with AI, MACS should be diagnosed in the presence of serum cortisol after the 1 mg-dexamethasone suppression test (F-1mgDST) above 1.8 µg/dL (50 nmol/L) and in the absence of clinically overt hypercortisolism. Unfortunately, the great majority of the available studies on the risk of fragility fractures, mainly vertebral fractures (VFx), in patients with AI did not use the same criteria for defining MACS as those proposed by the ESE-ENSAT guidelines (6). Even though recent studies showed that MACS is associated with an increased prevalence (8, 9) and incidence of VFx (8), data from intervention studies on the possible effect of the recovery from MACS on bone fragility are completely lacking. For these reasons, the recent ESE-ENSAT guidelines still consider that the association between MACS and osteoporosis is not yet well established (1).

Therefore, the aim of the present study was to evaluate the incidence of VFx in patients with AI with MACS after removal of the adrenal adenoma or during a conservative follow-up.

## Patients and Methods

### Subjects

This study includes 2 separate analyses of different cohorts.

Firstly, we reanalyzed retrospective longitudinal data obtained from a multicenter study, performed from January 2008 to June 2013, on the effect of surgery or conservative approach in patients with subclinical hypercortisolism (7). As per our protocols, at the time of that study, subclinical hypercortisolism was diagnosed in patients with AI and without signs and/or symptoms of cortisol excess and in the presence of F-1mgDST >5.0 μg/dL (138 nmol/L) or in the presence of ≥2 out of F-1mgDST >3.0 μg/dL (83 nmol/L), adrenocorticotroph hormone (ACTH) levels <10 pg/mL (2.2 pmol/L), and 24-hour urinary free cortisol (UFC) levels > 70 μg/24 hours (193 nmoL/24 hours). In all patients with AI and subclinical hypercortisolism we suggested surgery, explaining its possible advantages and disadvantages. Briefly, among 605 subjects consecutively referred for unilateral AI, on the basis of the inclusion and exclusion criteria, as reported in the original study (7), 55 patients were found to be affected by subclinical hypercortisolism. By applying the ESE-ENSAT criteria for diagnosing MACS (1), 53 of these subjects met the criteria and their data were reanalyzed: 31 patients (Study 1-Group A, 23 females) underwent surgery, while 22 patients who refused the surgical option were conservatively followed (Study 1-Group B, 10 females). All subjects signed the informed consent form before entering the study, which was approved by the ethics committees of the 2 centers where the study was conducted (Fondazione IRCCS Cà Granda, Milan, Italy; “Casa Sollievo della Sofferenza” Hospital IRCCS, San Giovanni Rotondo, Foggia, Italy)

The second part of the present study reports data from a prospective multicenter randomized protocol (Study 2) conducted in a cohort of patients with MACS who were recruited between September 2016 and February 2020 and randomized to surgery or to a conservative approach in order to evaluate the effect of adrenalectomy on several outcomes possibly associated with hypercortisolism, including variations of body weight, blood pressure, glucometabolic control, bone mineral density (BMD), and the number of incident VFx (NCT 04860180). Data on the changes of blood pressure and glucometabolic control after 6 months of follow-up have been recently published (4). Here we report the data on BMD and incident VFx after 24 months of follow-up.

The study was approved by the Milan Area B Ethics Committee (n. 809_2015, resolution n. 516 25/03/2016). Briefly, we included patients with (1) diagnosis by imaging of unilateral AI >1 cm with radiological features at computed tomography typical of adrenocortical adenoma; (2) age between 40 and 75 years. We excluded patients on the basis of the presence of (1) signs or symptoms specific of hypercortisolism and/or AI >5 cm and/or with radiologic appearance not consistent with an adrenocortical adenoma, or biochemical evidence of pheochromocytoma and aldosteronism, since these patients were directly referred to surgery (10); (2) incomplete data; (3) intake of drugs influencing dexamethasone metabolism and/or cortisol metabolism or secretion; (4) disorders or conditions potentially affecting bone health, such as renal and hepatic diseases, thyrotoxicosis, hypogonadism, alcoholism, hematological and rheumatologic disease, and eating disorders.

**Figure 1.**
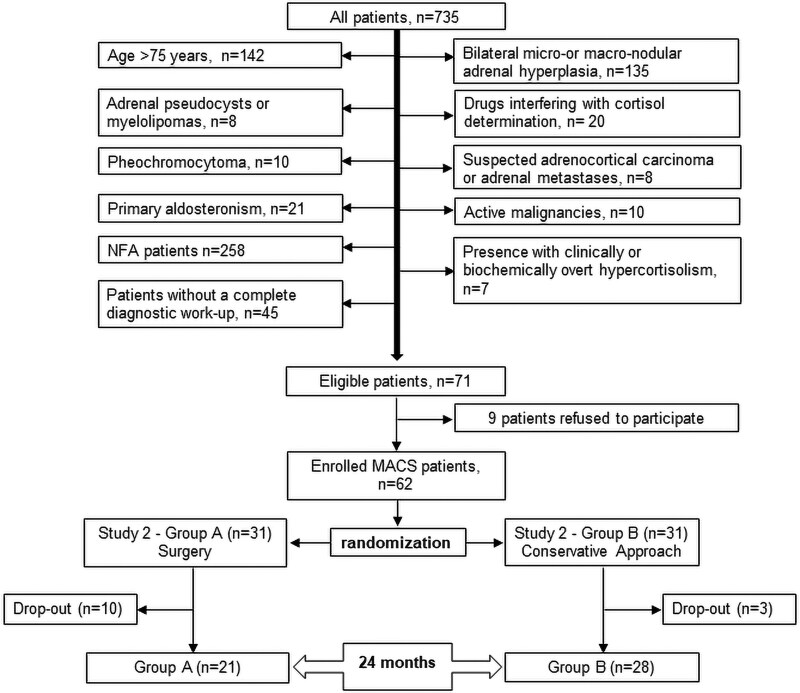
Enrollment procedure of study 2 (prospective randomized). Drop-out causes in Group A: consent withdrawal (n = 4), cancer occurrence (n = 1), death for COVID-19 (n = 1); lost at follow-up (n = 10). Drop-out causes in Group B: adrenalectomy for adenoma enlargement (n = 2); lost at follow-up (n = 1).

Figure 1 summarizes the enrollment procedure of Study 2. A total of 735 patients with AI have been evaluated for inclusion. After the exclusion of 399 patients on the basis of the above-mentioned inclusion and exclusion criteria, all patients with confirmed F-1mgDST levels between 1.8 and 5.0 µg/dL (50 and 138 nmol/L) were considered eligible for study inclusion. Among the 71 eligible patients, 9 refused to participate and 62 patients were enrolled: 31 were randomized to surgical intervention (Study 2-Group A) and 31 to conservative approach (Study 2-Group B). A block randomization was used to reduce bias and achieve balance in the allocation of participants to treatment arms. Eventually, 49 patients (21 in Study 2-Group A and 28 in Study 2-Group B) completed the 24-month study protocol. In Study 2-Group A, 4 patients withdrew consent before surgery, 1 patient was diagnosed with breast cancer after baseline assessments and dropped out the study, and 1 patient died of Coronavirus disease-2019 before surgery. In Study 2-Group B, 1 patient had a rapid adenoma size increase due to internal nodule bleeding after 5 months of follow-up and underwent adrenalectomy. All patients were informed of the purpose and nature of all procedures and gave informed consent

In both Study 1 and Study 2, AIs were discovered by imaging performed for unrelated diseases (ie, computed tomography scan, ultrasonography, or magnetic resonance); ultrasound findings were confirmed with computed tomography scan. At computed tomography, all adrenal masses were hypodense and homogeneous, consistent with the diagnosis of adrenocortical adenoma, and the contralateral adrenal gland was normal. Laparoscopic or laparotomic (open) adrenalectomy was performed and no patient had complications. In all patients the histological findings were consistent with adrenal adenoma. After adrenalectomy, precautionary steroid therapy with hydrocortisone was administered and, in all patients, cortisol secretion was re-evaluated, after 2 months, by the ACTH stimulation test. In patients with persistent adrenal insufficiency, hypothalamic–pituitary–adrenal function was reassessed every 6 months. The median duration of steroid substitutive therapy was 12 and 5 months in Study 1 and in Study 2, respectively.

In all subjects of both Study 1 and Study 2, BMD was measured by dual-energy X-ray absorptiometry (Hologic Discovery, Bedford MA, USA) at the lumbar spine (LS, precision 1.0%), total hip (TH, precision 1.8%) and femoral neck (FN, precision 1.8%) and expressed as g/cm^2^ and SD units (Z-score) in relation to reference population and as the change in Z-scores per year (ΔZ-score/year) between baseline and the end of follow-up. Lateral and anteroposterior spinal radiographs (T4-L4) were performed in all subjects. Two radiologists, who were blinded to hormonal and BMD data, independently reviewed the radiographs and discussed the questionable cases. Prevalent and incident VFx were diagnosed using semiquantitative visual assessment (11): fractures assessed on lateral T4-L4 spine radiographs were defined as reductions of more than 20% in anterior, middle, or posterior vertebral height. In order to increase specificity, we considered as VFx only vertebrae with at least moderate (>25% compression) deformity. Fractured vertebrae were excluded from BMD measurement. In both Study 1 and Study 2, type 2 diabetes was diagnosed using World Health Organization criteria (12).

### Methods

Serum and 24-hour urinary calcium, serum 25-hydroxyvitamin D (25OHVitD), phosphate, parathyroid hormone (PTH), and albumin levels were assessed. Calcium, albumin, and phosphate were measured by standard colorimetric techniques. Serum intact PTH and 25OHVitD concentration was measured by electrochemiluminescence immunoassay (reference interval 15-65 pg/mL [1.7-6.9 pmol/L] and 30-75 ng/mL [100-250 nmol/L], respectively). According to Italian guidelines (13), in the presence of hypovitaminosis D, patients were supplemented with cholecalciferol in order to achieve 25OHVitD levels above 30 ng/mL (75 nmol/L), and all patients with a dietary calcium intake <1000 mg/day were supplemented with calcium carbonate or calcium citrate.

In patients enrolled in Study 1, as per our protocols, calcium phosphate parameters were measured every 12 months. The Study 2 protocol contemplated measuring cortisol secretion and calcium phosphate parameters every 6 and 3 months, respectively, until the end of follow-up.

In all patients from Study 1-Group B on the basis of our national guidelines (14) alendronate was offered to 11 patients with a FRAX 10-year probability of a major osteoporotic fracture ≥10% (15). Out of the 7 patients who accepted the weekly bisphosphonate therapy, only 5 patients had an adherence ≥80%.

On the basis of our national guidelines (14), an antiresorptive therapy (alendronate or risedronate) was prescribed in 2 and 5 patients from Study 1-Group B and Study 2-Group B, respectively. No patient from Study 1-Group A and from Study 2-Group A was treated with bone active drugs as per our protocol (16).

### Statistical Analysis

In both Study 1 and Study 2, the results are expressed as mean ± SD or median (interquartile range, IQR) as appropriate. Categorical variables were compared by χ^2^ test or Fisher exact test as appropriate. Comparison of continuous variables among the different groups was performed using the Student t test for paired or unpaired data, as appropriate, for normally distributed variables and using the Mann–Whitney U test or the Wilcoxon signed-rank test, as appropriate, for not normally distributed variables.

Logistic regression analysis was used in Study 1 to assess the association between the occurrence of incident VFx after adjusting for adrenalectomy (yes/no) and for the independent variables known to be associated with increased fracture risk (ie, age, gender, F-1mgDST levels, LS BMD, and VFx at baseline) as well as other variables that were found to be different between surgically and not surgically treated patients at baseline. This analysis was repeated also including the use of bone active drugs (as independent variable) in the model.

Statistical analysis was performed by SPSS version 28.0 statistical package (IBM Corporation, Armonk, New York, United States). *P* values less than .05 were considered statistically significant.

## Results

### Study 1: Retrospective Data

Clinical characteristics of patients surgically (Study 1-Group A) and conservatively treated (Study 1-Group B) at the beginning and at the end of follow-up are reported in Table 1. In both groups, serum calcium, creatinine, phosphorous, and PTH levels and 24-hour urinary calcium levels were within the normal range and did not change between baseline and the end of follow-up (data not shown). The number of patients with prevalent VFx was not different between the 2 groups. The VFx were severe (ie, > 40% compression deformity) in 7 and 3 patients, in Study 1-Group A and Study-1 Group B, respectively

**Table 1.** Retrospective study (study 1): comparison of clinical and biochemical characteristics between surgically treated (study 1-group a) and conservatively treated (study 1-group B) MACS patients at baseline and at the end of follow-up

	Study 1-Group A (n = 31)			Study 1-Group B (n = 22)		
	Baseline	End of FU	*P*	Baseline	End of FU	*P*
Age (yrs)	63 (57-67)	65.7 (60.5-70.6)	.10	64 (61-72)	66 (64-75)	.20
Gender (females)	23*^a^* (74.2)	—	—	10 (45.5)	—	—
BMI (kg/m^2^)	26.0 (24.5-29.1)	26.4 (23.7-29.6)	.93	25.2 (23.3-29.6)	25.6 (23.9-31.1)	.41
Duration of FU (months)	—	36*^c^* (24-48)	—	—	27 (24-44)	—
Diameter of adenoma (cm)	3.2 (2.2-4.0)	—	—	2.5 (2.1-3.5)	—	—
25-Hydroxyvitamin D (ng/mL)	20.7 (14.7-26)	38 (34.4-42)	.001	20.5 (16.3-28.8)	36.4 (33.8-43.2)	.001
ACTH (pg/mL)	7.2 (5.0-9.1)	20.6*^d^* (12.8-46.6)	.001	8.4 (5.9-9.0)	8.9 (7.7-9.4)	.26
F-1mgDST (μg/dL)	3.6 (2.4-5.9)	.9*^d^* (.7-1.0)	.001	3.4 (2.2-4.3)	3.1 (2.6-5.8)	.04
UFC (μg/24 hour)	68 (41-96)	25*^d^* (20-50)	.001	56.1 (33.2-81.4)	56.3 (30.1-56.3)	.72
L1-L4 BMD (Z-score)	−0.81*^a^* (−2.0-0.1)	−0.54 (−1.3-0.4)	.29	0.2 (−1.4-1.2)	0.4 (−1.5-1.4)	.96
L1-L4 ΔZ-score/year	—	0.10 (−0.05-0.2)	—	—	−0.01 (−0.01-0.1)	—
Femoral neck BMD (Z-score)	−0.7*^b^* (−1.2-0.1)	−0.4 (−1.0-0.0)	.44	0.0 (−0.6-0.5)	−0.2 (−0.4-0.7)	.89
Femoral neck ΔZ-score/year		0.05 (−1.05-0.74)			−0.5 (−1.7-1.2)	
Total hip BMD (Z-score)	−0.8*^b^* (−1.4-0.1)	−0.5 (−0.8-0.4)	.21	0.05 (−0.8-0.5)	0.05 (−0.6-0.7)	.69
Total hip ΔZ-score/year		0.05 (−0.05-0.15)			−0.01 (−0.05-0.2)	
Patients with prevalent VFx (%)	14 (45.2)	—	—	14 (63.6)	—	—
Patients with incident VFx (%)	—	3*^e^* (9.7)	—	—	11 (50.0)	—
Patients with type 2 diabetes (%)	5*^a^* (16.1)	5*^c^* (16.1)	1.00	9 (40.9)	9 (40.9)	1.00

Data are median (interquartile range) or absolute number with percentage in parenthesis.

To convert to SI units multiply ×: ACTH 0.22, UFC 2.756, cortisol 27.56, 25-hydroxyvitamin D 2.5.

Abbreviations: ΔZ-score/year, change of Z-score per year between baseline and end of FU; ACTH, adrenocorticotroph hormone; BMD, bone mineral density; BMI, body mass index; F-1mgDST, serum cortisol levels after 1-mg dexamethasone suppression test; FN, femoral neck; FU, follow-upLS, lumbar spine; MACS, mild autonomous cortisol secretion was diagnosed in presence of F-1mgDST >1.8 µg/dL; UFC, urinary free cortisol; TH, total hip; VFx, vertebral fractures.

^*a*^ *P* < .005 and

^*b*^ *P* < .05 vs conservatively treated patients at baseline.

^*c*^ *P* < .05,

^*d*^ *P* < .001, and

^*e*^ *P* < .005 vs conservatively treated patients at the end of FU.

At baseline, age, BMI, adenoma size, 25OHD, ACTH, 1mg-DST, and UFC levels and prevalence of hypertension were not different between the 2 groups, while LS and FN BMD and prevalence of type 2 diabetes was lower in Study 1-A than in Study 1-B group.

Compared with Study 1-Group A, Study 1-Group B showed an increased incidence of VFx at the end of the follow-up. In Study 1-Group B, BMD at both LS and total hip (TH) was comparable between baseline and the end of follow-up, while F-1mgDST levels were slightly higher at baseline than at the end of follow-up. A new VFx (7 moderate and 4 severe) occurred in 11 (50%) of the patients, among whom 2 patients were treated with bisphosphonates and 5 patients had a prevalent VFx.

In Study 1-Group A, BMD at both LS and FN did not change between baseline and the end of follow-up. All 3 patients in this group with incident VFx (all moderate, 9.4%) had a prevalent VFx at baseline.

Considering the whole sample of the Study 1, patients with incident VFx and those without incident VFx were comparable as far as the F-1mgDST levels at baseline were concerned (data not shown). In the whole sample of patients, the logistic regression analysis showed that the surgical treatment of MACS was significantly associated with a 6.8-fold reduced risk of a new VFx (odds ratio [OR] 0.147, 95% CI 0.023-0.924, *P* = .041), but not with age, gender, duration of follow-up, degree of hypercortisolism (as expressed by F-1mgDST), LS BMD (as expressed as Z-score), and presence of VFx and of type 2 diabetes at baseline (Table 2). The results were confirmed even after adjusting for the use of bone active drugs (data not shown)

**Table 2.** Independent associations between the occurrence of an incident vertebral fragility fracture and adrenalectomy, age, gender, duration of follow-up, degree of hypercortisolism, lumbar spine bone mineral density, and presence vertebral fracture and of type 2 diabetes at baseline in patients with MACS

	aOR	95% CI	*P* value
Adrenalectomy (yes vs no)	0.147	0.023-0.924	**<.041**
Age (1-year increase)	1.061	0.919-1.225	.417
Gender (women)	0.971	0.167-5.659	.818
Duration of follow up (1 month increase)	0.994	0.943-1.048	.632
F-1mgDST (1 µg/dL increase)	0.649	0.280-1.508	.315
LS-BMD (1 Z-score increase)	1.139	0.579-2.240	.707
Prevalent vertebral fracture at baseline (yes vs no)	0.551	0.119-2.538	.444
Type 2 diabetes mellitus (yes vs no)	0.547	0.091-3.274	.251

Statistically significant associations are in bold.

Abbreviations: aOR, odds ratio adjusted for the variables included in the model; BMD, bone mineral density; F-1mgDST, 1-mg dexamethasone suppression test; LS, lumbar spine; MACS, mild autonomous cortisol secretion.

### Study 2: Randomized Prospective Study

Clinical characteristics of patients in Study 2-Group A (surgically treated) and in Study 2-Group B (conservatively treated) at baseline are reported in Table 3. The 2 groups were not different in terms of demographic characteristics (age, sex, BMI), and in terms of cortisol secretion parameters (F-1mgDST, UFC, ACTH). The BMD measured at spine, FN, and TH was not different between the 2 groups at baseline as well as the prevalence of VFx. Two patients and no patients had severe VFx in Study 2-Group B and in Study 2-Group A, respectively.

**Table 3.** Randomized prospective study (Study 2): comparison of clinical and biochemical characteristics between surgically treated (Study 2-Group A) and conservatively treated (Study 2-Group B) patients with MACS at baseline

	Study 2-Group A (n = 21)	Study 2-Group B (n = 28)	*P* value
Age (yrs)	63 (56.5‒72.5)	69 (61‒73)	.19
Women, n (%)	14 (67)	22 (78)	.35
BMI (kg/m^2^)	27.7 (24.4‒31.1)	26 (23.3‒30.6)	.37
Diameter of adenoma (cm)	3.2 (2.7‒3.8)	2.9 (2.3‒3.4)	.1
ACTH (pg/mL)	8.8 (5.9‒11.5)	9 (5.7‒12.3)	.74
F-1mgDST (μg/dL)	3.5 (2.3‒4.5)	2.5 (2.2‒3.6)	.10
UFC (μg/24 hour)	27.1 (16.5‒40.9)	22 (12.6‒29.1)	.11
Patients with type 2 diabetes (%)	5 (23.8)	5 (17.9)	.37
L1-L4 BMD (Z-score)	0.15 (−0.57-0.75)	0.1 (−0.7-0.9)	.99
Femoral neck BMD (Z-score)	−0.55 (−0.30-0.37)	−0.1 (−1.1-0.4)	.72
Total hip BMD (Z-score)	0.28 (−0.6-0.9)	−0.05 (−0.57-0.82)	.67
Patients with prevalent VFx (%)	4 (21)	6 (21.4)	.97

MACS was diagnosed in presence of F-1mgDST >1.8 µg/dL (50 nmoL/L) Data are median (interquartile range) or absolute number with percentage in parenthesis.

To convert to SI units multiply ×: ACTH 0.22, UFC 2.756, cortisol 27.56.

Abbreviations: ΔZ-score/year, change of Z-score per year between baseline and end of FU; ACTH, adrenocorticotroph hormone; BMD, bone mineral density; BMI, body mass index; F-1mgDST, serum cortisol levels after 1-mg dexamethasone suppression test; FU, follow-up; MACS, mild autonomous cortisol secretion; UFC, urinary free cortisol.

**Figure 2.**
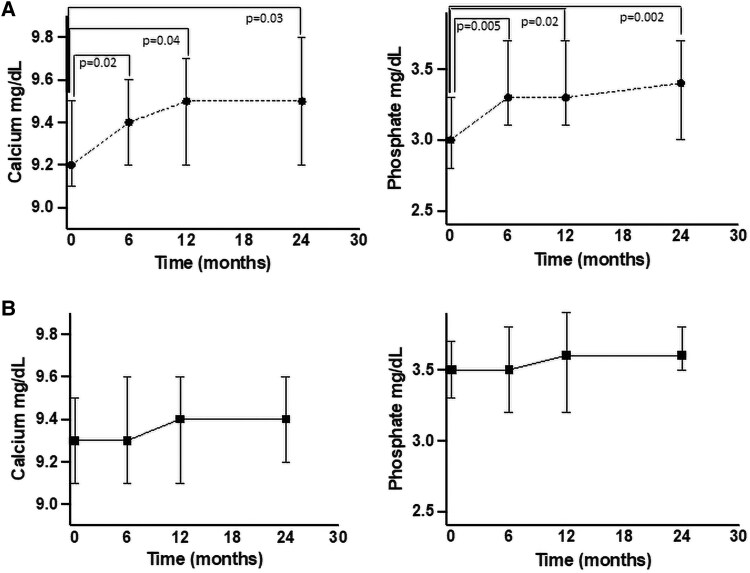
Study 2 (prospective randomized): changes in calcium and phosphate levels from baseline to end of follow-up in surgically treated subjects (group A) and in conservatively treated ones (group B). In surgically treated patients, calcium and phosphate levels significantly increased from baseline to 6 months, 12 months, and 24 months. No statistically significant changes in phosphate levels were found in conservatively managed patients (Groups B) from baseline to 6, 12, and 24 months.

The patients were followed over time (Table 4). Regarding patients who underwent surgery (Study 2-Group A), after 24 months of follow-up, we observed a normalization of F-1mgDST levels and, as expected, a significant increase in ACTH values (<.001). Furthermore, in Study 2-Group A we observed an increase in calcium and phosphorus levels between baseline and 6 months, 12 months, and the end of follow-up (Fig. 2).

**Table 4.** Randomized prospective study (Study 2): comparison of clinical and biochemical characteristics between surgically treated (Study 2-group A) and conservatively treated (Study 2-Group B) patients with MACS at baseline and at the end of follow-up

	Study 2-Group A (n = 21)			Study 2-Group B (n = 28)		
	Baseline	24 months of follow-up	*P* value	Baseline	24 months of follow-up	*P* value
BMI (kg/m^2^)	27.7 (24.4-31.1)	27.7 (23.1-31.8)	.88	26 (23.3-30.6)	25.2 (22.9-27.5)	.52
ACTH (pg/mL)	8.8 (5.9-11.5)	25.9 (17.3-44.3)	<.001	9 (5.7-12.3)	10.6 (8.3-14.4)	.13
F-1mgDST (μg/dL)	3.5 (2.5-4.5)	0.8 (0.59-1.45)	<.001	2.5 (2.2-3.6)	2.8 (2.1-4.2)	.57
UFC (μg/24 hour)	27.1 (16.5-40.9)	26.5 (16.6-41.6)	.78	22 (12.6-29.1)	22.2 (14.3-32.8)	.35
Calcium (mg/dL)	9.2 (9.1-9.5)	9.5 (9.2-9.8)	.03	9.3 (8.9-9.5)	9.4 (9.2-9.6)	.56
Phosphate (mg/dL)	3.0 (2.8-3.4)	3.4 (3.0-3.7)	.04	3.5 (3.3-3.7)	3.6 (3.5-3.8)	.17
25-hydroxyvitamin D (ng/mL)	27.8 (19-36)	32.9 (29.1-37.7)	.15	33.2 (26.7-43.5)	35.9 (32.2-43.0)	.40
24 hours urinary calcium (mg/kg/day)	1.35 (1.01-2.53)	1.51 (0.95-2.08)	.71	2.10 (1.30-2.97)	1.43 (0.79-2.92)	.29
Alkaline phosphatase (U/L)	62.5 (52.0-77.5)	66 (53-86)	.53	69 (60.5-74.5)	69 (57-81)	.78
L1-L4 BMD (Z-score)	0.2 (−0.6-0.8)	0.2 (−0.3-1.0)	.55	0.1 (−0.7-0.9)	0.3 (−0.6-1.3)	.38
Femoral neck BMD (Z-score)	−0.5 (−0.3-0.4)	−0.2 (−0.4-0.3)	.72	−0.1 (−1.1-0.4)	−0.3 (−0.9-0.5)	.92
Total hip BMD (Z-score)	0.2 (−0.6-0.9)	0.3 (−0.3-0.7)	.78	−0.1 (−0.6-0.8)	0 (−0.7-1.1)	.80
Patients with incident VFx (%)	—	1 (4.8)*^a^*		—	7 (25)	.04

Data are median (interquartile range) or absolute number with percentage in parenthesis. Data are mean ± SD with range in parenthesis or absolute number with percentage in parenthesis.

To convert to SI units multiply ×: ACTH 0.22, UFC 2.756, cortisol 27.56, 25hydroxyvitamin D 2.5.

Abbreviations: ΔZ-score/year, change of Z-score per year between baseline and end of FU; ACTH, adrenocorticotroph hormone; BMD, bone mineral density; BMI, body mass index; F-1mgDST, serum cortisol levels after 1-mg dexamethasone suppression test; FN, femoral neck; FU, follow-up; LS, lumbar spine; MACS, mild autonomous cortisol secretion was diagnosed in presence of F-1mgDST >1.8 µg/dL; TH, total hip; UFC, 24-hour urinary free cortisol.

^*a*^ *P* < 0.05 vs conservatively treated patients at the end of FU.

In patients who were followed up with clinical observation (Study 2-Group B), no significant changes were observed in cortisol or ACTH levels, or in calcium and phosphorus levels when comparing baseline values to those after 24 months (Fig. 2). BMD remained stable in both groups over the 24-month follow-up period.

Finally, the fragility fracture incidence was evaluated in all patients. After a 24-month follow-up, a total of 8 patients had an incident fragility fracture. All incident fractures were morphometric VFx and occurred in 1 patient (moderate VFx) in the surgical group (Study 2-Group A) and in 7 patients (3 severe VFx) in the conservatively treated group (Study 2-Group B) (*P* = .04), including the 2 subjects treated with bisphosphonates. Among the 8 patients with incident VFx, 5 patients had a VFx at baseline (1 and 4 in Study Group A and Study 2-Group B, respectively). The F-1mgDST levels at baseline were not different between patients with incident VFx and those without incident VFx (data not shown). The surgical treatment of MACS was significantly associated with a 4.5-fold reduced risk of new VFx (OR 0.22, 95% CI 0.07-0.71) even after adjusting for age (OR 0.65, 95% CI 0.05-0.84)

## Discussion

**Figure 3.**
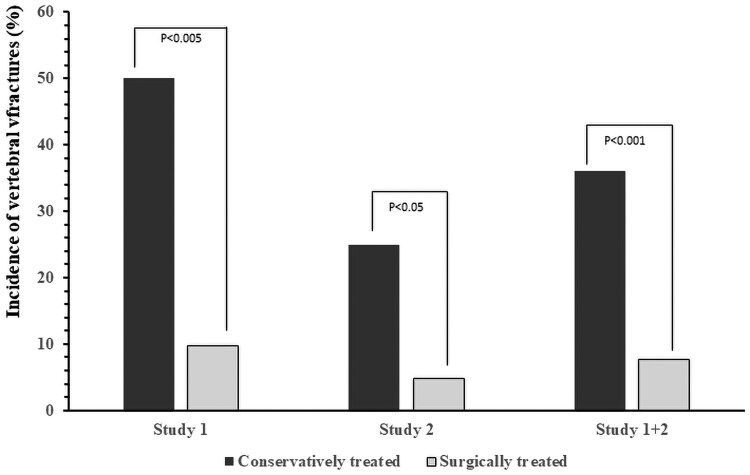
Incidence of vertebral fractures in conservatively treated patients and surgically treated patients from Study 1, Study 2 and from Study 1 plus Study 2 taken together. Study 1: retrospective longitudinal study. Study 2: prospective randomized study. For each comparison the difference between conservatively treated subjects and surgically treated subjects is statistically significant. As compared with conservatively treated patients, in Study 1, Study 2, and Study 1 + 2 surgically treated subjects have a 6.8-fold, 4.5-fold, and 6.5-fold reduced risk of fragility fractures, respectively.

This is the first study evaluating the effect of the recovery from MACS on bone health and in particular on the risk of fragility fracture. In both the retrospective and randomized prospective studies, we found that subjects who underwent the removal of the adrenal adenoma causing MACS had an important VFx risk reduction, while conservatively treated patients maintained a high rate of VFx incidence (Fig. 3). Moreover, during follow-up, BMD did not significantly change in both surgically and conservatively treated patients.

The finding that, in patients with MACS, recovery from mild cortisol excess leads to a dramatic reduction of VFX risk is in line with previous studies showing the benefit of surgery in fracture risk reduction in patients with subclinical hypercortisolism (6, 7, 17), a condition of asymptomatic cortisol excess diagnosed in the past with criteria different from those currently used to define MACS (1). However, although recent studies suggested a negative effect even of MACS on bone health and in particular on the risk of fragility fractures (8, 9, 17), data showing the effect of surgery on fracture risk were not available so far. Overall, considering together data from the retrospective and prospective study, we found that in patients with MACS surgical treatment is associated with a 6.5-fold reduced risk of VFx. The finding that patients with AI and MACS are at increased risk of fragility fracture and that this risk could be reduced by the removal of the adrenal mass is important since it suggests that bone fragility could be considered among the criteria for addressing the treatment of choice in these patients.

As in patients with subclinical hypercortisolism, even in patients with MACS, the fracture risk, though importantly reduced, is not totally abolished by surgery. This could be related to different reasons. Firstly, even though in surgically treated patients the follow-up period started after the withdrawal of steroid substitutive therapy, we cannot exclude that this therapy could have been excessive for bone health, at least in some patients. Moreover, the length of recovery from sarcopenia may have played a role in slowing down fracture risk reduction after surgery (6, 17, 18). Importantly, in both studies all surgically treated patients with MACS who experienced an incident VFx had a prevalent VFx at baseline and, therefore, might themselves be at increased risk of subsequent VFx (19). Finally, although all patients with a dietary calcium intake below 1000 mg/day were supplemented with calcium carbonate or calcium citrate, it is not possible to exclude that the adherence to calcium supplements has been inadequate in some patients. This is important because after glucocorticoid treatment, an adequate calcium intake is crucial for bone health recovery (16), which, in patients withdrawing from glucocorticoids, is partially compensated by the increase of calcium and phosphate absorption by the gut (20). It is of note that in the prospective randomized study, calcium and phosphate levels increased in surgically treated patients but not in conservatively treated ones, thus confirming the beneficial effect on bone of the recovery from even mild hypercortisolism.

A further significant result is that the randomized study confirms that the conservatively treated patients with MACS are exposed to an increased risk of VFx, similarly to what has already been suggested from retrospective data (8, 9). Unfortunately, the present studies were not designed to provide information regarding the predictive factors for incident fragility fractures in patients with MACS managed with a conservative approach. However, considering together the retrospective and prospective data of the present study, among the 18 conservatively managed patients who had an incident VFx, 11 subjects already had a prevalent VFx at baseline, confirming that the presence of a prevalent VFx should always be considered as an important risk factor for a subsequent fracture (19). Additionally, the present studies do not show a possible role of F-1mgDST levels in predicting the risk of fractures in patients with MACS managed with a conservative approach. However, looking at the association between the mean F-1mgDST levels with the incidence of VFx in the cohorts of conservatively treated patients in the present randomized prospective study (2.5 µg/dL and 25%, respectively), in a previously published (8) retrospective observational study (3.0 µg/dL and 36.4%, respectively) and in the present retrospective study (3.5 µg/dL and 50.0%, respectively), a possible predictive role of F-1mgDST levels on the risk of incident fractures could be hypothesized. This is in line with the results of a large observational cross-sectional study showing a direct association between F-1mgDST levels and VFx prevalence in patients with MACS (9).

Some findings of the present studies were somewhat expected. The fact that fragility fractures were only at vertebral sites is in line with previous studies on both subclinical hypercortisolism and MACS (6, 17), and it could be explained by the fact that trabecular bone is highly sensitive to glucocorticoid excess (21). A further expected finding is that the prevalence of patients with an inadequate response to bisphosphonates (4 out of 7 patients, 57.1% considering the 2 studies together) is higher than that expected in postmenopausal osteoporosis (22), confirming that MACS confers an additional risk of fragility fractures. Finally, even the present studies confirm that the absence of a low BMD at baseline and/or of a BMD reduction during the follow-up could not be considered a safe parameter in evaluating the fracture risk in patients with MACS, as already described in patients with subclinical hypercortisolism (23). Indeed, it is widely accepted that BMD evaluation is not entirely reliable for predicting the fracture risk in glucocorticoid induced osteoporosis since bone quality, which is not captured by BMD assessment, plays an important role (24). Therefore, in the clinical evaluation of patients with MACS, other tools, beside BMD (eg, trabecular bone score [TBS]), should be studied in order to identify patients at higher risk of incident fractures (23). Indeed, particularly in patients with secondary osteoporosis, the fracture risk strongly depends on, beside BMD, the alteration of bone quality, which includes bone geometry (bone size, shape), bone macro- and microarchitecture (eg, connectivity and thickness of trabeculae, thickness and porosity of cortical bone), the balance and rate of bone remodeling, bone mineralization, and the type and organization of collagen or other components of the bone matrix (25). Thus, it is possible to hypothesize that, as described in patients with overt and mild cortisol excess (26, 27), in patients with MACS, TBS, a dual-energy X-ray absorptiometry–derived surrogate marker of bone quality, could add information on the risk of fragility fractures, regardless of BMD.

The lack of the availability of TBS data has to be considered a first limitation of these studies. Secondly, due to its retrospective design, we cannot exclude that in Study 1 some confounding factors could have exerted a role. For instance, the higher disease activity in conservatively managed patients (as mirrored by F-1mgDST levels) may have impacted on the fracture risk. However, the fact that the association between surgical treatment and fracture risk reduction was independent of F-1mgDST levels renders this hypothesis less likely. Similarly, we did not record data on tobacco use, which is thought to be associated with an increased fracture risk (28). Thirdly, due to the need for steroid substitutive therapy, the observation period in the surgically treated patients lasted for a mean of 12 months more than that in the conservatively managed subjects. However, the shorter follow-up in conservatively treated subjects should have decreased rather than increased the rate of VFx. In addition, the use of bone turnover markers, which were not included in both Study 1 and Study 2 determinations, could have been informative, particularly for the prediction of patients with MACS at higher risk of incident VFx (29). Moreover, radiologists were not mandatory blinded for surgical intervention or not, and, thus, we cannot exclude that they could have been influenced by awareness of the patient's status. Finally, the prevalence of 53 patients with MACS out of 605 patients with AI included in the retrospective study may appear too low considering that many data show a prevalence of MACS in AI of up to 50% (1-3). However, at the time of the study, only patients with subclinical hypercortisolism were recommended to undergo adrenalectomy and were systematically followed for both skeletal and extraskeletal complications. Therefore, in the present study, we could report data only from the 53 patients with subclinical hypercortisolism who also met the MACS criteria, as data on skeletal complications were available only for these patients.

Notwithstanding these limitations, this study is important since it shows for the first time with both retrospective and randomized prospective approaches that subjects undergoing the removal of the adrenal adenoma causing MACS have an important VFx risk reduction, while conservatively treated subjects maintain a high rate of VFx incidence. Further larger studies should be designed in order to identify MACS patients at higher risk of incident VFx. In the meantime, while approaching patients with MACS, physicians should take into consideration the risk of VFx in patients who do not undergo surgery.

## Data Availability

All datasets generated during and/or analyzed during the current study are not publicly available but are available from the corresponding author on reasonable request.

## Clinical Trial Information

ClinicalTrials.gov Identifier: NCT04860180 (registered February 16, 2021).

## Abbreviations

25OHVitD: 25-hydroxyvitamin D
ACTH: adrenocorticotroph hormone
AI: adrenal incidentaloma
BMD: bone mineral density
F-1mgDST: cortisol after 1-mg dexamethasone suppression test
FN: femoral neck
LS: lumbar spine
MACS: mild autonomous cortisol secretion
PTH: parathyroid hormone
TBS: trabecular bone score
TH: total hip
UFC: urinary free cortisol
VFx: vertebral fractures
